# Allogeneic Adipose-Derived Mesenchymal Stem Cell Transplantation Alleviates Atherosclerotic Plaque by Inhibiting Ox-LDL Uptake, Inflammatory Reaction and Endothelial Damage in Rabbits

**DOI:** 10.3390/cells12151936

**Published:** 2023-07-26

**Authors:** Yanhong Li, Guiying Shi, Wei Liang, Haiquan Shang, Huiwu Li, Yunlin Han, Wenjie Zhao, Lin Bai, Chuan Qin

**Affiliations:** NHC Key Laboratory of Human Diseases Comparative Medicine, National Human Diseases Animal Model Resource Center, Beijing Key Laboratory for Animal Models of Emerging and Remerging Infectious Diseases, Institute of Medical Laboratory Animal Science, Chinese Academy of Medical Science (CAMS) & Comparative Medicine Centre, Peking Union Medical College (PUMC), Beijing 100021, China

**Keywords:** adipose-derived stem cells, atherosclerosis, rabbits, inflammation, exosomes

## Abstract

Atherosclerosis (AS) is a chronic inflammatory disease of arteries fueled by lipids. It is a major cause of cardiovascular morbidity and mortality. Mesenchymal stem cells have been used for the treatment of atherosclerotic lesions. Adipose-derived stem cells (ADSCs) have been shown to regulate the activation state of macrophages and exhibit anti-inflammatory capabilities. However, the effect of allogeneic ADSCs in the treatment of AS have not been investigated. In this study, the early treatment effect and preliminary mechanism analysis of allogeneic rabbit ADSCs intravenous transplantation were investigated in a high-fat diet rabbit model. The polarization mechanism of rabbit ADSCs on the macrophage was further analyzed in vitro. Compared with the model group, blood lipid levels declined, the plaque area, oxidized low-density lipoprotein (ox-LDL) uptake, scavenger receptor A1 and cluster of differentiation (CD) 36 levels were all significantly reduced, and the accumulation of inflammatory M1 macrophages, apoptosis, interleukin (IL)-6 and tumor necrosis factor (TNF)-α expression were decreased. The endothelial cells (CD31), M2 macrophages, IL-10 and the transforming growth factor (TGF)-β levels increased. In vitro, ADSCs can promote the M1 macrophage phenotypic switch toward the M2 macrophage through their secreted exosomes, and the main mechanism includes increasing arginase 1 expression and IL-10 secretion, declining inducible nitric oxide synthase (iNOS) expression and TNF-α secretion, and activating the STAT6 pathway. Therefore, allogeneic rabbit ADSC transplantation can transmigrate to the aortic atherosclerotic plaques and show a good effect in lowering blood lipids and alleviating atherosclerotic plaque in the early stage of AS by inhibiting ox-LDL uptake, inflammatory response, and endothelial damage.

## 1. Introduction

Atherosclerosis (AS) is a chronic inflammatory disease of arteries. The pathophysiological mechanisms of AS are very complex, and mainly involve accumulation of lipids in the subendothelial space, inflammatory responses of vascular endothelial cells (ECs), and transformation of macrophages into foam cells induced by oxidized low-density lipoprotein (ox-LDL), cell apoptosis, necrosis and fibrosis [1,2,3,4,5]. Subendothelial accumulation of macrophages at sites of endothelial dysfunction and intimal lipoprotein retention are pivotal steps in AS [6]. Macrophages can drive atherogenesis in the chronic inflammatory processes [6]. Inflammation is initiated by the retention of ApoB (apolipoprotein B)-containing lipoproteins in the arterial wall [7]. Both inflammatory M1 and regulatory M2 macrophages are found in atherosclerotic lesions. M1 macrophages contribute to inflammation by secreting pro-inflammatory cytokines such as interleukin (IL)-6 and tumor necrosis factor (TNF)-α after the intake of modified LDL. M2 macrophages have the function of resisting inflammation, immunomodulation, promoting the clearance of apoptotic cells and tissue repair by efferocytosis and releasing cytokines such as IL-10 and transforming growth factor (TGF)-β [8,9]. Cholesterol accumulation in macrophages occurs following ox-LDL uptake by scavenger receptors (SRs), including cluster of differentiation (CD) 36 and SR-A1 [10].

AS is a leading cause of mortality despite the availability of effective treatment for reducing serum lipids [5]. Mesenchymal stem cells (MSCs) can repair many different types of tissue damage and defects [6,11], and have emerged as a promising tool for the treatment of atherosclerotic cardiovascular disease [12,13,14]. Intravenous infusion of MSCs for multiple atherosclerotic lesions was shown to contribute to the remodeling of the vasculature [15,16]. Confirmation of the anti-inflammatory and anti-apoptotic properties of MSCs is a key imperative [17,18,19,20]. Studies have demonstrated that adipose-derived stem cells (ADSCs) possess immunomodulatory abilities and can regulate the activation state of macrophages [21,22]. Furthermore, ADSCs were shown to exhibit superior anti-inflammatory capabilities, phagocytic activity, and anti-apoptotic capability compared with bone-marrow-derived stem cells (BMSCs) [23]. Similar to other stem cells, ADSCs have the capability to differentiate into different cell lineages, such as ECs, cardiomyocytes, smooth muscle cells, adipocytes, and neurons [24]. Currently, there is a paucity of studies that have investigated the effect of ADSCs in the treatment of different stages of AS. We have earlier reported the effect of umbilical cord mesenchymal stem cells (UCSCs) in relieving aortic atherosclerotic plaque in the treatment of AS [25]. In the present study, we evaluated the therapeutic effect of intravenous transplantation of ADSCs derived from rabbit adipose tissue in the early stage of AS and performed preliminary mechanism analysis in a rabbit model. We found that allogeneic rabbit ADSC transplantation had a good effect in lowering blood lipids, relieved the inflammatory response in aortic atherosclerotic plaques and enhanced anti-inflammatory effects by regulating the production of macrophages, ECs, and inflammation-related factors. Our findings underline the broader therapeutic prospects for the clinical application of ADSCs, and also provide a basis for clinical screening of more suitable treatment strategy for patients with AS.

## 2. Materials and Methods

### 2.1. Animals

New Zealand rabbits (2.5–3.5 months old; 50/50 sex distribution) were purchased from Beijing Fu Long Teng Fei Experimental Animal Research Institute Co., Ltd. (Beijing, China). (SCXK [Jing] 2018-0009). The breeding environment of the rabbits is the same as previously described [25]. The rabbits were randomly divided into four groups: normal (normal diet + phosphate-buffered saline, PBS), model (high-fat diet + PBS), normal diet control (NDC; normal diet + ADSCs) and treatment (T; high-fat diet + ADSCs). Random numbers were generated using a computer based on the standard = RANDBETWEEN () function in Microsoft Excel. There were 3 female and 3 male rabbits in each group. Animal experiments were approved by the Institutional Animal Care and Use Committee (IACUC) of the Institute of Medical Laboratory Animal Science, CAMS&PUMC (QC19020). In the experiments, for short-term pain, gentle operation, appropriate massage, or improvement of the environment (toys to regulate emotions, etc.) can be performed; if the pain is severe, analgesics can be administered appropriately to relieve it.

### 2.2. Rabbit ADSC Culture and Induced Differentiation Identification

Adipose tissues (approximately 25 mL) were extracted from inguinal regions of the rabbits and cut into small tissue blocks rinsed with PBS (14190250, GIBCO) to remove residual blood. Subsequently, the tissue blocks were collected in centrifuge tubes, and an equal volume of 0.1% collagenase I (K05541-100 mg, Merck, Darmstadt, Germany) was added for digestion at 37 °C for 30 min. The digested cells were collected and a proper amount of an α-MEM complete medium (12571071, GIBCO) was added, containing a 10% fetal bovine serum (FBS) (10099141C, GIBCO) and a 1% Penicillin–Streptomycin (P/S) (15140122, GIBCO), then placed into a 6-well plate, and cultured with a 5% CO_2_ at 37 °C. The culture solution was changed every three days until the cells achieved confluence; further, the cells were digested with pancreatin (25200056, GIBCO) and passaged.

Induced differentiation experiments: (1) ADSCs were induced to differentiate into adipocytes; from the well growing ADSCs at the 3rd generation, ADSCs were collected and inoculated in a 24-well plate, 10^4^/well, 37 °C, 5% CO_2_. When the cells achieved a more than 80% confluence, the medium was removed and 2 mL of adipogenic induction medium (RBXMD-90031, Santa Clara, CA, USA) was added. The induction medium was changed every 3 days, for about 21 days. The control group was supplemented with an α-MEM complete medium. Then, the cells were stained with oil red O liquid (G1262, Solarbio, Beijing, China): first, the induction medium was removed and washed twice with PBS, fixed with a 4% paraformaldehyde for 30 min, then washed twice with a 60% isopropanol; the oil red O staining solution was added for 60 min; after that, PBS was added to wash 3 times. The cells were observed and images were captured with an inverted microscope (LWD200-371, Cewei). (2) ADSCs were induced to differentiate into bone cells: cells with the same conditions as above were added into an osteogenic induction medium (RBXMD-90021, Cyagen) and cultured for 21 days. The cells were stained with the Alizarin Red solution (G1452, Solarbio): the induction medium was removed and the cells washed, fixed with a 4% paraformaldehyde for 30 min; then, the Alizarin Red staining solution was added for 5 min; the staining solution was discarded and the cells were washed 3 times with PBS. The cells were examined under an inverted microscope and images were captured.

### 2.3. Animal Model Construction and ADSC Transplantation Therapeutic Schedule of AS 

The detailed method for constructing a high-fat diet rabbit model is described elsewhere [25]. ADSCs were transplanted into treatment and normal diet control rabbits by intravenous injection into the ear vein after 1 month of a high-fat diet, and the dose was 6 × 10^6^ in 500 μL of PBS once every 2 weeks lasting for 3 months [26,27]. The same amount of PBS liquid was injected into the animals of model and normal groups under the same conditions and in the same manner. In animal experiments, different personnel were responsible for different parts, including grouping and feeding personnel, ADSC transplantation personnel, dissection and testing personnel (unaware of the grouping), data collection and analysis personnel. The animals were treated with ADSCs or measured according to animal serial number. In the experiments, the animals were monitored when they had abnormal conditions, such as when they were unable or extremely slow to move, had difficulty in ingesting food and water, and continued to be anorexic, and the frequency of monitoring was 1–2 times per day. During the experiments, there was no abnormality in the animals, and they were included in the experimental evaluation as planned.

### 2.4. Detection of Blood Lipids and Other Related Biochemical Indicators

In the 1st and 3rd month after the treatment, blood samples were collected and tested in accordance with the method described before [25]; the main indices measured were triglyceride (TG), total cholesterol (TC), low-density lipoprotein cholesterol (LDL-C), alanine aminotransferase (ALT), Apolipoprotein (APO) A1, APOB, and creatine kinase MB (CK-MB) levels (Beckman AU5800, Bria, CA, USA).

### 2.5. Analysis of Ultrasonic Evaluation of Vascular Plaque 

Plaque formation in the carotid artery, aortic arch, and abdominal aorta in T, model, and normal group animals were detected by the color Doppler ultrasound diagnostic system (SIEMENS, ACUSON SC2000) in the 1st and 3rd month of modeling.

### 2.6. Histopathological Analysis of Aorta

As reported before [25], at the end of the experiment, the animals were euthanized with Zoletil^TM^50 (20 mg/kg, Intramuscular injection) and inguinal artery bleeding. Aortas were collected and atherosclerotic plaques were detected with oil red O staining, and aortic plaque area percentage was measured. Histopathological examination of aorta was performed by Hematoxylin-eosin (HE) staining, as well as immunohistochemical staining for ADSC makers CD90 (66766-1-Ig, Proteintech, Wuhan, China) and CD73 (Ys4834R, Yaji Biological, Shanghai, China); macrophage marker CD68 (ab955, Abcam, Hong Kong), arginase 1 (bs-23837R, Bioss, Woburn, MA, USA) and iNOS (bs-2072R, Bioss); cytokines IL-6 (bs-6312R, Bioss), TNF-a (bs-2150R, Bioss), IL-10 (bs-0698R, Bioss) and TGF-β (bs-4538R, Bioss); ECs marker CD31 (ab199012, Abcam); proliferating antigen Ki67 (ab15580, Abcam); ox-LDL (TS2004R, Yaji Biological); and scavenger receptors SRA1 (bs-6763R, Bioss) and CD36 (bs-8873R, Bioss). The reagents and experimental methods were the same as those in the previous studies [25,28,29]. The methods are roughly as follows: Paraffin sections were dewaxed to water; then, the antigen was retrieved by a microwave, the endogenous peroxidase activity was eliminated with hydrogen peroxide, the non-specific antigen was blocked with goat serum; following this, the primary antibodies were added at 4 °C overnight. The next day, the secondary antibodies were added, and staining was performed with a DAB staining solution. The slices were immersed in a hematoxylin solution to stain the nucleus, dehydrated and cleared, covered with coverslips. In addition, terminal deoxynucleotidyl transferase mediated dUTP nick end-labeling (TUNEL) staining (S7101, Sigma-Aldrich, St. Louis, MO, USA) of apoptotic cells was performed in each group. TUNEL testing was performed according to the instructions. After staining, the sections were examined and photographed using a light microscope (BX51, Olympus, Tokyo Japan).

### 2.7. Study of ADSCs on Macrophage Polarization In Vitro

Human acute leukemia cells THP-1 (TIB-202TM) were purchased from the American Type Tissue Collection (ATCC) and cultured with RPMI 1640 (RPMI Medium 1640 (t) 25 mM HEPES, 72,400047, Gibco, Carlsbad, CA, USA) containing a 10% FBS and a 1% (P/S) at 37 °C in a humidified incubator with a 5% CO_2_. Then, the THP-1 cells were transferred to a 10 mL sterile centrifuge tube; the cells were centrifuged and the supernatant was discarded. The cell suspension (5 × 10^5^/L) was diluted, phorbol 12-myristate 13-acetate (PMA, P1585, Sigma) (final concentration: 20 ug/L) was added, mixed well, and transferred to a 6-well plate for incubation, induced into macrophages. After 48 h, the original culture medium was replaced by an RPMI1640 medium containing a 5% FBS and a 1% (P/S), and then the induced macrophages and the medium were transferred to the Transwell plates (3450, Corning, Corning, NY, USA). ADSCs and their culture medium were added to the incubate system and co-cultured for 48 h. The cell culture groups were as follows: Control group (untreated macrophages); Model group (macrophages + LPS (100 ng/mL, L2880, Sigma) + INF-γ (20 ng/mL, 300-02, peprotech)); Treatment group (macrophages + LPS (100 ng/mL) + INF-γ (20 ng/mL) + ADSCs). 

Culture supernatants were collected for detecting the cytokine TNF-α level secreted by M1 macrophages and the IL-10 level from M2 macrophages with the ELISA kit (JL10208, j&l biological) and (EH0173, FineTest) according to the manufacturer’s protocol. The cells were fixed in a 4% paraformaldehyde (E672002, Sangon Biotech), washed with PBS, blocked with serum for 30 min, incubated overnight with anti-arginase 1 (bs-23837R, Bioss) and iNOS (bs-0162R, Bioss) antibodies at 4 °C. The fluorescence-labeled secondary antibody was added for 30 min at room temperature (RT). Nuclei were stained by DAPI (D9542, Sigma). Fluorescence signals were observed with a fluorescence microscope (Leica, Wetzlar, Germany). 

The cells were lysed in a RIPA buffer (P0013B, Beyotime) and protein was extracted. Equal amounts of proteins were resolved on SDS-PAGE gels and transferred onto PVDF membranes (V273606, Bio-rad). After blocking with a 5% bovine serum albumin in a Tris-buffered saline containing a 0.1% Tween 20 (P9416, Sigma), the membranes were incubated with anti-STAT6 (bsm-52400R, Bioss) antibodies at 4 °C overnight, followed by incubation with secondary antibodies for 1 h at RT. The relative expression of the target protein was measured with the ratio of the optical density of the target protein to the internal reference protein.

### 2.8. The Isolation of ADSC-Derived Exosomes and Their Effect on Macrophage Polarization

ADSC-derived exosomes were extracted by ultracentrifugation. The cultured ADSC supernatant was centrifuged at 2000× *g*, 4 °C for 10 min; the obtained supernatant was centrifuged for 30 min, at 4 °C, 10,000× *g*. Then, the supernatant was transferred to an ultra-high-speed centrifuged tube, and centrifuged at 110,000× *g*, 4 °C for 75 min. The supernatant was discarded, the pellet was resuspended in PBS and filtered with a 0.22 μm membrane (ISEQ00010, Millipore, Burlington, MA, USA). The samples were transferred to an ultracentrifuge tube at 4 °C, 110,000× *g*, and centrifuged for 75 min again. The exosomes were resuspended in PBS, and the presence and morphological characteristics were verified by transmission electron microscopy (TEM) (JEN-1400, Japan); markers CD9 (EXOAB-CD9A-1, SBI), HSP70 (ab2787, abcam), TSG101 (EXOAB-TSG101-1, SBI) and Calnexin (YT01613, Immunoway, Plano, TX, USA) were detected by Western blot, and protein concentration was quantified by the Pierce BCA protein Assay kit (23227, Thermo, Waltham, MA, USA). The THP-1 cells were induced into the adherent macrophages by exposure to PMA (20 ug/L) for 48 h, and then LPS (100 ng/mL), INF-γ (20 ng/mL) and ADSC-derived exosomes (5 ug/mL) were added for co-culture for 48 h. The effect of ADSC-derived exosomes on M1 polarization of macrophages was investigated by detection of related secreted factors, marker proteins and signaling molecules of M1 and M2 macrophages.

### 2.9. Statistical Analysis

Continuous variables were presented as mean values ± standard deviation of the mean. Image-pro-plus software (Media cybernetics, Inc. IPP 6.0, Rockville, MD, USA) and Image J 1.42 (NIH, Bethesda, MD, USA) were used for quantitative analyses of histopathological and cytological studies. Microsoft Excel 2010 (Microsoft Corporation, Redmond, WA, USA) was used for data recording and statistical analyses. Intergroup differences were assessed using Student’s *t*-test. *p* values < 0.05 were considered indicative of statistical significance.

## 3. Results

### 3.1. Morphological Characteristics and Induced Differentiation Identification of ADSCs

**Figure 1:**
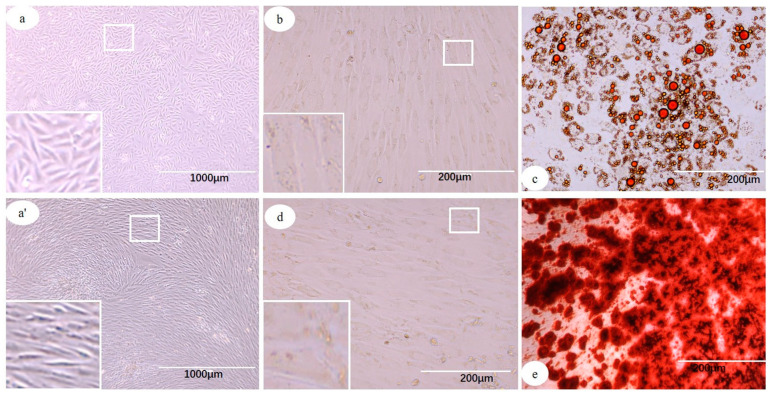
Morphology and induced differentiation identification of ADSCs. (**a**,**a′**) ADSCs showing spindle morphology, and arranged in radial or whirlpool shape. (**b**,**c**) ADSCs cultured in adipogenic medium and stained with oil red O showing plump and oblate cytoplasm, with presence of large and small lipid droplets (**c**), (**b**) control. (**d**,**e**) ADSCs cultured in osteogenic medium and stained with Alizarin Red showing a large number of crimson calcified nodules and presence of osteocytes (**e**), (**d**) control.

ADSCs at P3 showed spindle shape morphology, vigorous growth, and were arranged in a radial or a whirlpool shape (Figure 1a,a’). ADSC-induced lipogenesis experiment showed changes in cell morphology, with a plump and oblate cytoplasm. After staining with oil red O, large or evenly distributed small lipid droplets were observed (Figure 1b,c). Induced osteogenesis experiment showed small nodules in the center or edge of cell growth, and after staining with Alizarin Red, a large number of crimson calcified nodules were found (Figure 1d,e). 

### 3.2. ADSC Transplantation Decreases the Levels of Serum Lipid in AS Rabbits

**Figure 2:**
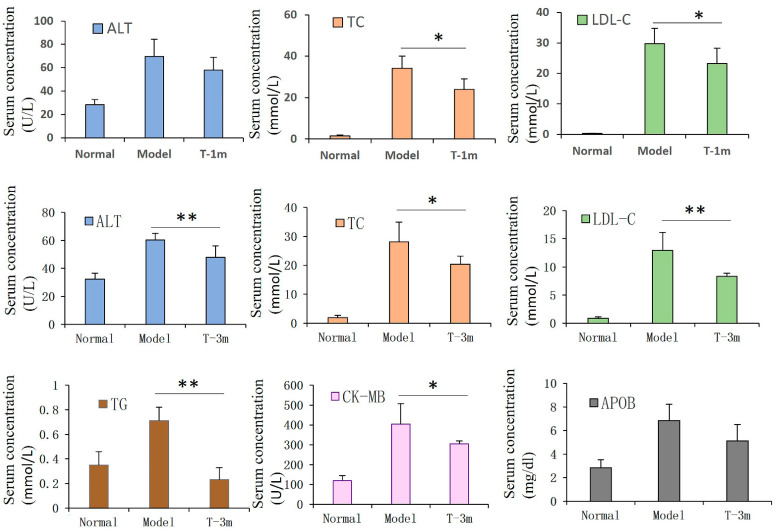
Serum index alteration after transplantation of ADSCs in AS rabbits. After transplantation of ADSCs in the early stage of AS, serum TC and LDL-C levels at 1 month and ALT, LDL-C, TC, TG, and CK-MB levels at 3 months in ADSC groups were significantly lower than those in model group. Student’s *t*-test was performed for comparing statistical differences between groups. (n = 6 in each group). * *p* < 0.05, ** *p* < 0.01 compared with the model group.

After 1 month of ADSC transplantation, the serum TC (*p* < 0.05) and LDL-C (*p* < 0.05) levels in the ADSC group were significantly lower than those in the model group. At the 3rd month of ADSC transplantation, serum ALT (*p* < 0.01), LDL-C (*p* < 0.01), TC (*p* < 0.05), TG (*p* < 0.01), and CK-MB (*p* < 0.05) levels in the ADSC group were significantly lower than those in the model group (Figure 2). 

### 3.3. ADSC Transplantation Alleviates Atherosclerotic Plaque Formation in AS Rabbits

**Figure 3:**
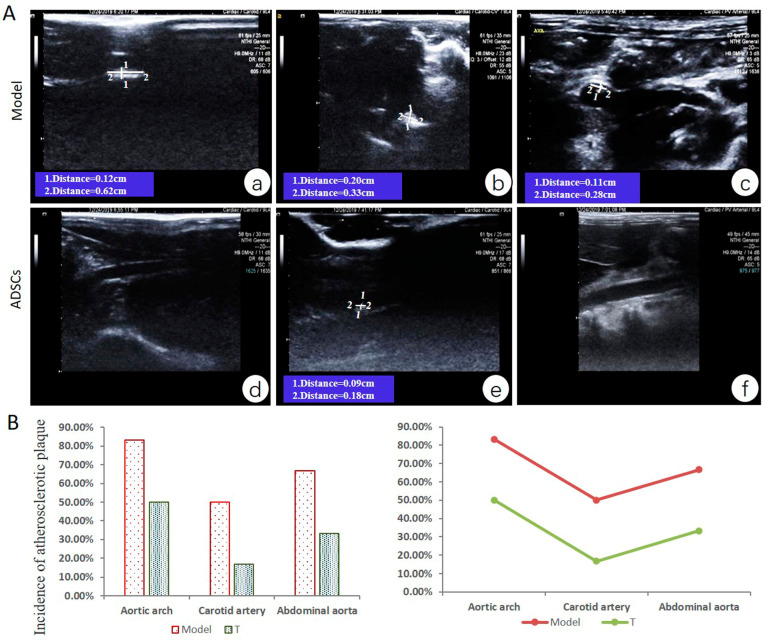
Arterial plaque formation after ADSC transplantation at early stage of AS. (**A**) (**a**). Long strip plaque (width, 0.12 cm; length, 0.62 cm) in carotid artery with thickened intima; (**b**). Plaque (width, 0.20 cm; length, 0.33 cm) at aortic arch; (**c**). Plaque (width, 0.11 cm; length, 0.28 cm) in the abdominal aorta; (**d**). Normal carotid artery wall; (**e**). Small plaque (width, 0.09 cm; length, 0.18 cm) in the middle of the abdominal aorta; (**f**). Normal abdominal aorta. (**B**) The incidence of atherosclerotic plaque in aortic arch, carotid artery, and abdominal aorta (incidence = number of animals with plaques/total number of animals in each group × 100%, n = 6 in each group).

Vascular ultrasonic detection showed no signs of atherosclerotic plaques in the aorta, carotid artery, and abdominal aorta of animals at the first month of high-fat diet. At the third month of modeling, the aortic arch, carotid artery, and abdominal aorta of model group animals showed different degrees of atherosclerotic plaque (incidence: 83.3% (5/6 animals), 50% (3/6 animals), and 66.7% (4/6 animals), respectively). After treatment for 2 months with ADSCs, the animals in the T group also had atherosclerotic plaque (incidence in the aortic arch, carotid artery, and abdominal aorta were 50% (3/6 animals), 16.7% (1/6 animals), and 33.3% (2/6 animals), respectively) (Figure 3). 

### 3.4. ADSC Transplantation Reduces the Size of Aortic Atherosclerotic Lesions in Rabbits

**Figure 4:**
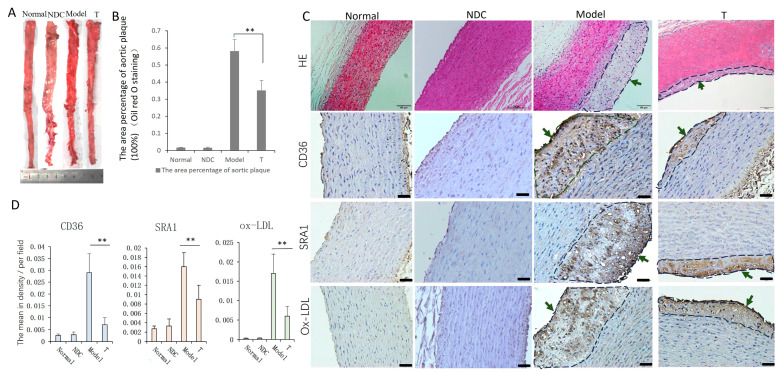
Oil red O staining and detection of ox-LDL, SRA-1 and CD36 in aorta. (**A**,**B**) Analysis of Oil red O-stained aorta: the plaque area percentage of T group was lower than that of the model group. (**C**) Pathological analysis of aorta showing different degrees of lipid deposit and plaque formation (green arrows) in the model and treatment groups, bar = 100 µm. (**D**) Ox-LDL and macrophage scavenger receptors SRA-1 and CD36 in aorta showed significant decrease in the T group compared with that in the model group, respectively, bar = 50 µm (n = 5–6 in each group). ** *p* < 0.01.

On gross pathology examination, the area percentage of aorta atherosclerotic plaque in the model group was found to be significantly larger than that in the T group (*p* < 0.01) (Figure 4A,B). Histopathological analysis of HE-stained sections revealed different degrees of lipid deposition, intimal thickening, and inflammatory cell infiltration in the aortas of the model and treatment groups. The aortic plaque in the T group was relatively small (Figure 4C). The absorption of ox-LDL (*p* < 0.01) and the expression of macrophage scavenger receptors SRA-1 (*p* < 0.01) and CD36 (*p* < 0.01) in the aortic tissues of the T group were significantly reduced compared with those of the model group (Figure 4C,D). 

### 3.5. ADSC Transplantation Inhibits Inflammatory Reaction of Aorta in AS Rabbits

**Figure 5:**
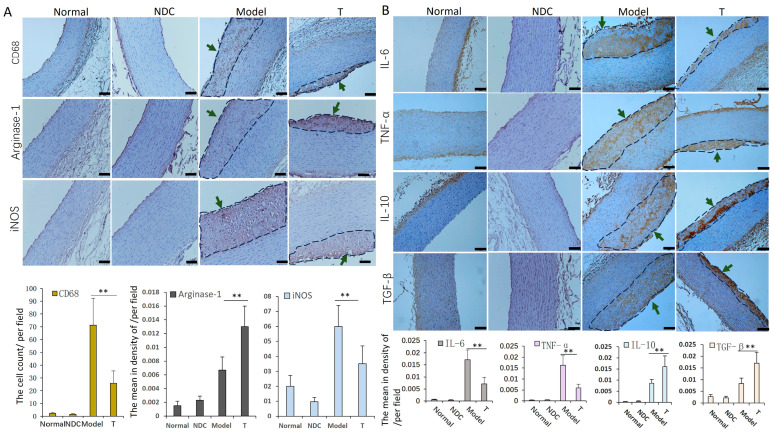
Detection of inflammatory cells and cytokines in aorta. Staining and analysis of macrophage marker molecules CD68, iNOS and Arginase-1 (**A**), inflammatory factors TNF-α and IL-6, and anti-inflammatory factors IL-10 and TGF-β (**B**): the levels of CD68, iNOS, IL-6 and TNF-α were lower, and the levels of IL-10 and TGF-β were higher in the T group compared with those in the model group, respectively (the areas of atherosclerotic plaque were circled with dashed lines, and indicated by green arrows) (n = 5–6 in each group). ** *p* < 0.01, bar = 100 µm.

The expression levels of macrophage marker molecules CD68 and iNOS, as well as inflammatory factors TNF-α and IL-6, declined (*p* < 0.01) in the T group compared with those in the model group, respectively. The levels of macrophage marker Arginase-1, and anti-inflammatory factors IL-10 and TGF-β were relatively increased (*p* < 0.01) in the ADSCs treatment group compared with those in the model group, respectively (Figure 5). 

### 3.6. ADSCs Can Promote Macrophage Phenotypic Switch toward M2 Subtype In Vitro

**Figure 6:**
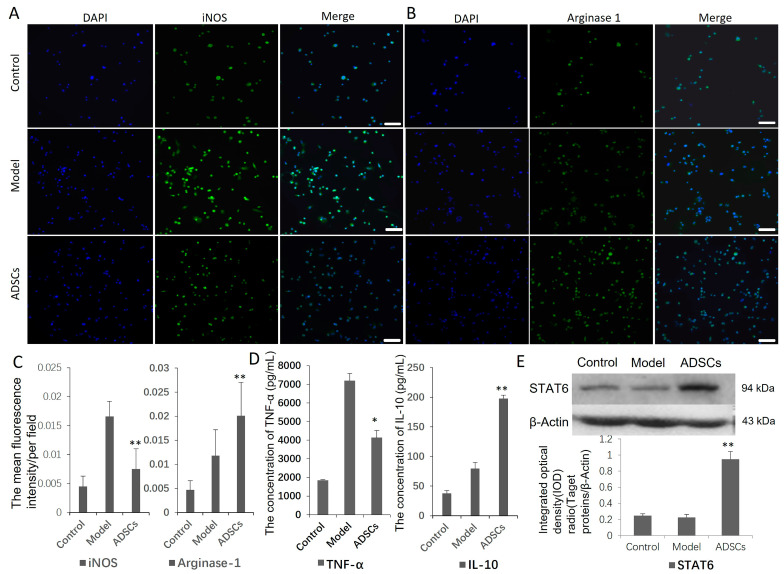
ADSC and macrophage polarization assay in vitro. (**A**–**C**) Immunofluorescence detection of M1, M2 macrophage marker proteins iNOS and arginase-1. Compared with the model group, iNOS level was lower, and arginase-1 expression was higher in ADSC treatment group. Original magnification is 200. (**D**) ELISA results suggested that level of cytokine TNF-α secreted by M1 macrophages decreased in ADSCs group. (**E**) Western blot detection showed the expression of STAT6 signal molecules increased significantly in ADSCs group. * *p* < 0.05, ** *p* < 0.01.

Co-culture ADSCs and induced M1 macrophage in vitro, iNOS expression (*p* < 0.01) and TNF-α secretion (*p* < 0.05) decreased, arginase 1 expression (*p* < 0.01) and IL-10 secretion (*p* < 0.01) increased, and the levels of STAT6 were up-regulated (*p* < 0.01) compared with those in the model group (Figure 6). 

### 3.7. ADSCs Promote Macrophage Phenotypic Switch toward M2 Subtype by ADSC-Derived Exosomes

**Figure 7:**
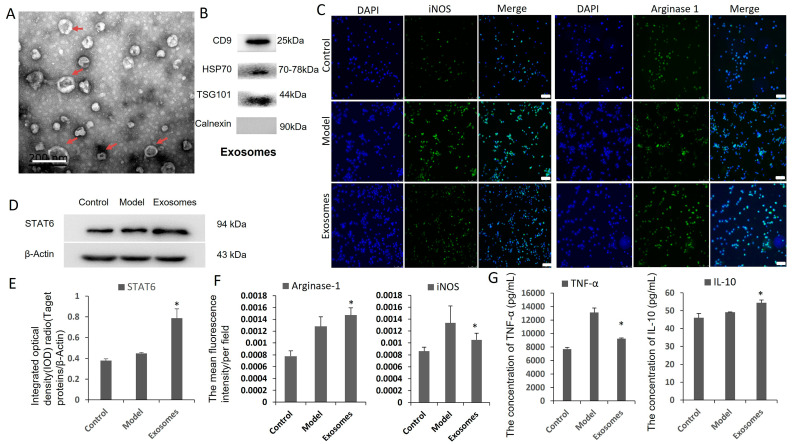
ADSC-derived exosomes and macrophage polarization assay in vitro. (**A**) TEM analysis presented that ADSC-derived exosomes were round membrane-bound vesicles and the diameter is about 30 mm to 150 nm as indicated by the red arrow. Scale bar is 200 nm. (**B**) Exosomes from rabbit ADSCs were identified by markers CD9, HSP70, TSG101 and Calnexin by Western blot. (**C**,**F**) Immunofluorescence detection of M1, M2 macrophage marker proteins iNOS and arginase-1. Compared with the model group, iNOS level was lower, and arginase-1 expression was higher in ADSC exosome treatment group. Original magnification is 200. (**D**,**E**) Western blot detection showed the expression of STAT6 signal molecules increased significantly in ADSC-derived exosome group. (**G**) ELISA results suggested that concentration of cytokine TNF-α decreased and IL-10 increased in ADSC exosomes group. * *p* < 0.05.

To elucidate the mechanism by which ADSC–macrophage cross-talk, ADSC-derived exosomes were extracted and co-cultured with the induced M1 macrophage in vitro. It was found that exosomes were nearly round membrane-bound vesicles with a 30–150 nm diameter (Figure 7A), and the extracted concentration is 0.21 μg/μL. Western blot showed positive for exosome markers CD9, HSP70, TSG101 and negative for control marker Calnexin (Figure 7B). In the ADSC-derived exosome treatment group, the iNOS expression (*p* < 0.05) and TNF-α secretion (*p* < 0.05) decreased, arginase 1 expression (*p* < 0.05) and IL-10 secretion (*p* < 0.05) increased, and the levels of STAT6 were up-regulated (*p* < 0.05) compared with those of the model group (Figure 7C–G). 

### 3.8. ADSC Transplantation Inhibits Apoptosis and Promotes EC Proliferation in AS Rabbits

**Figure 8:**
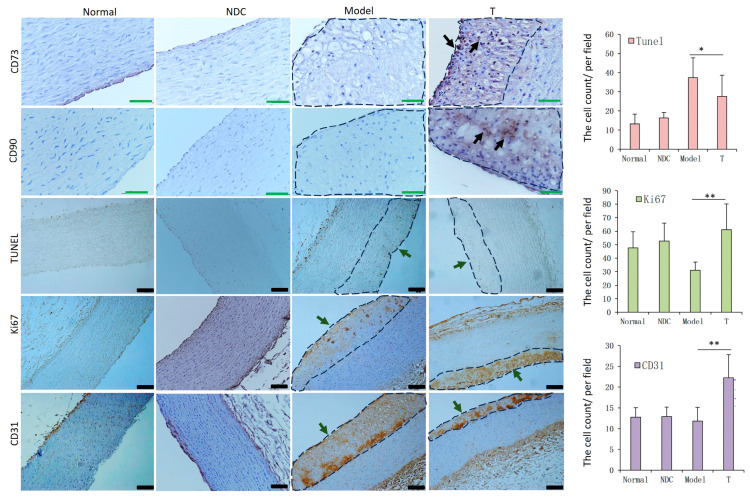
Detection results of ADSCs, apoptosis and proliferation in aorta. ADSCs locating staining using CD73 and CD90 showed that they mainly existed in the atheromatous plaque of the aorta (Black arrow), bar = 50 µm. Apoptosis (TUNEL assay) of arterial cells decreased, and expression levels of the cell proliferation molecule Ki67 and EC marker antigen CD31 increased in the T group compared to those in the model group, respectively (the areas of atherosclerotic plaque were circled with dashed lines), bar = 100 µm (n = 5–6 in each group). * *p* < 0.05, ** *p* < 0.01.

The location of ADSCs in the aorta was analyzed with markers CD90 and CD73, which revealed that ADSCs mainly existed in the atherosclerotic plaque of the aorta in the treatment group. In the detection of apoptosis and proliferation of aortic cells, we found a significant decrease in the apoptosis of arterial cells (*p* < 0.05) and a significant increase in the expression levels of cell proliferation molecule Ki67 (*p* < 0.01) and EC marker molecule CD31 (*p* < 0.01) in the treatment group compared with those in the model group, respectively (Figure 8). 

## 4. Discussion

Cell therapy is a contemporary research hotspot and it is already being used widely for the treatment of various diseases including AS. In this study, we show that allogeneic rabbit ADSC transplantation can reduce the development of the atherosclerotic lesion in the early-stage intervention. Fan et al. [30] reported that ADSC transplantation alleviated the pathological signs of aortic AS in rat models, and the ADSCs were isolated from C57BL/6J mice. A high-fat diet AS model (cholesterol-fed rabbits) [31] was used for our study, and its unique characteristics are discussed elsewhere [25]. ADSCs used in our experiment were derived from rabbit adipose tissue. From an immunological perspective, allotransplantation is perhaps a better choice than xenotransplantation [32]. Autologous and allogeneic transplantation of MSCs is a relatively safe method used for treatment of many diseases, and allogeneic cells can be prepared before disease onset which facilitates rapid administration [33]. Studies have demonstrated the distinct advantage of ADSCs in allotransplantation therapy compared with other cell sources [34]. Owing to their immunomodulatory capability, they are particularly suitable for allogeneic transplantation for therapeutic purposes [32]. Other reasons for the selection of ADSCs were that the yield of stem cells derived from the adipose tissue is higher than the yield of stem cells derived from peripheral blood [35], and ADSCs derived from the adipose tissue show a stronger proliferative potential than bone marrow derived stem cells [36]. Furthermore, adipose tissue is much easier to obtain than other tissues [24]. Therefore, in our study, allogeneic rabbit ADSC transplantation was used for treating AS, and the therapeutic effect was evaluated at an early stage. The results indicate that ADSCs have a good effect in lowering blood lipids and can relieve aortic plaque in AS. In our study, the main mechanism of the anti-atherosclerotic effect of ADSCs was by reducing blood lipid, inhibiting inflammation, and repairing damaged endothelium. This study can provide a certain theoretical basis and guiding significance for the clinical application of ADSC transplantation in AS treatment.

MSCs have the capacity to home and integrate into the injured and inflamed sites [37]. Studies have shown that after the injection of MSCs (including ADSCs) in mice, most cells become trapped in the lungs and 99% of cells are cleared from the circulation within 5 min [38,39]. MSCs have also been shown to have the ability to migrate to atherosclerotic plaques [40,41]. Another study found that MSCs primarily accumulate in the lungs after intravenous injection, and only a few migrate to the aorta [42]. In our previous study, we demonstrated that UCSCs can transmigrate to the vessel wall and were mainly observed in atherosclerotic plaques [25]. Therefore, ADSCs may have a similar fate to that of the MSCs mentioned above. In this study, we found that the ADSCs can also transmigrate to the aortic wall and into the aortic plaques. However, further in vivo experiments are required to determine the specific fate of ADSCs in blood and other organs after intravenous injection.

AS is a complex chronic inflammatory disease, and macrophages play a key role in the initiation and progression of AS. Foam cells are a hallmark feature of atherosclerotic plaques that develop due to the exposure to and the uptake of ox-LDL by macrophages [23,43,44]. As inflammation develops, the dead macrophages and foam cells form the necrotic lipid-filled cores, and the substances released from these cells have a toxic effect on cells contributing to plaque destabilization [23]. In this study, atherosclerotic plaques were observed in the aortic intima of the model group and ADSCs group; however, there was a significant decrease in the plaque area, the number of macrophages and the apoptotic cells in the treatment group. We also detected the expression of ox-LDL in the plaque and the levels of receptors CD36 and SRA1 which mediate the absorption of ox-LDL; they were also decreased in the T group. These changes can reduce the accumulation of macrophages and the formation of foam cells to some extent, thereby alleviating inflammation and AS progression.

In our study, the levels of the M1 macrophage maker iNOS and the pro-inflammatory cytokines TNF-α and IL-6 in the treatment group were decreased compared with those in the model group, while the levels of the M2 macrophage maker Arginase-1, inflammatory suppressors IL-10 and TGF-β were increased. IL-10 can inhibit macrophage activation and pro-inflammatory cytokine expression [40]. It has been shown that TNF-α and IL-6 are mainly secreted by M1 macrophages, while TGF-β and IL-10 are mainly produced by M2-type macrophages [8,9]. These showed that ADSCs can reduce the accumulation of inflammatory M1 macrophages and increase the level of anti-inflammatory M2 macrophages in aortic plaques. In vitro experiments further confirmed that ADSC intervention increased the level of the M2-type macrophage marker molecule arginase-1 and promoted the secretion of IL-10, declined the level of the M1-type macrophage marker molecule iNOS and inhibited the secretion of inflammatory cytokine TNF-α [45]. It is reported that the M2 phenotype macrophages can be promoted by STAT6 pathway, and TNF-α was reduced in parallel by reduced NF-κB expression [46]. Furthermore, in our experiments, ADSCs enhanced STAT6 activation in macrophages. Therefore, ADSCs can promote the phenotypic transformation of macrophages from M1 to M2 by the activation of the STAT6 pathway. We further evaluated the effect of ADSC-derived exosomes on macrophage polarization by the same experiments, and the results confirmed that the potential mechanism of ADSC function may be through their secreted exosomes. Therefore, regulating the polarization of macrophages via ADSC-derived exosomes may be another mechanism by which ADSCs inhibit inflammation.

In addition, as discussed previously [25], serum LDL, TG, TC, and APOB concentrations play a role in the pathogenesis and development of atherosclerotic lesions. In our study, serum TC and LDL-C levels decreased after 1 month and 3 months of early ADSC transplantation, while TG and CK-MB levels decreased after 3 months of early treatment. These data suggest that ADSC transplantation at an early stage may potentially alleviate AS by reducing serum lipids. Fan et al. [30] also reported that ADSC transplantation decreased the serum levels of TC, TG, and LDL-C in AS rats, which is consistent with our experimental results. It is reported that the activation of Kupffer cells was reduced, and further VLDL metabolism was inhibited in MSC-treated mice [41]. Kupffer cells, the resident macrophages of liver [47], can promote the secretion of VLDL in hepatocytes through the expression mediators [48]. VLDL can further metabolize to LDLs by hepatic tissues [49]. Down-regulation of TNF-α can reduce VLDL synthesis [50], and up-regulation of IL-10 can reduce plasma cholesterol, mostly due to a reduced VLDL in LDLR^−/−^mice [51]. Therefore, these results showed that MSCs can reduce dyslipidemia by decreasing inflammation in AS [5]. It is reported that the delivery of gingiva-derived mesenchymal stem cells led to a significant decrease in TG, TC, and LDL in hyperlipidemic mice, which may occur through affecting the expression of hepatic genes associated with lipid metabolism [52]. In our study, we found that ADSCs can inhibit inflammation by regulating the aggregation and apoptosis of macrophages, production of inflammatory factors (TNF-α, etc.) and anti-inflammatory factors (IL-10, etc.). Therefore, ADSC treatment can achieve a certain effect on lowering blood lipids, which may be through inhibiting inflammation. Further experiments will explore other possible mechanisms.

In the cell proliferation detection, the levels of proliferating cells and ECs in the treatment group were more than those in the model group. The effect of ADSC transplantation in inducing recovery of ECs is critical for alleviating AS, because the injury and decrease of ECs can greatly promote the progression of AS [53]. Studies have shown that the transplanted ADSCs can differentiate into vascular endothelial cells and play an essential role in injury repair through the contribution to angiogenesis [54]. Therefore, another potential mechanism by which ADSC transplantation may relieve AS is by promoting cell proliferation and repairing damaged ECs. It has been reported that ADSC-derived exosomes protect ECs against AS by restraining the expression of AS-associated miR-324-5p [55]; another report suggested that ADSCs inhibit vascular inflammation and EC dysfunction in AS rats through the inhibition of the NF-κB signaling pathway [30]. In our study, we confirmed that ADSCs can inhibit inflammation by activating the STAT6 signaling pathway through their secreted exosomes. 

## 5. Conclusions

This study mainly revealed that allogeneic rabbit ADSC transplantation in the early stage of AS can have a good effect on lowering blood lipids and alleviating the progress of atherosclerotic plaque lesions. The preliminary mechanism by which ADSC transplantation alleviates AS is partially similar to that of UCSCs, and mainly includes reducing the serum lipids, inhibiting inflammation and apoptosis, inhibiting ox-LDL absorption and receptor SRA-1, CD36 expression, and repairing damaged endothelium. However, this study focuses on the analysis of the molecular mechanism of ADSCs playing an anti-inflammatory role, and found that it is mainly through activating the STAT6 pathway by their secreted exosomes to inhibit the inflammatory polarization of macrophages. This study mainly evaluated the therapeutic effect and analyzed the preliminary mechanism of allogeneic rabbit ADSC transplantation on AS. The next step will increase the number of animals, analyze the fate of the injected ADSCs and the effect and mechanism on intestinal flora, dynamic changes in ADSCs and immune cells in target organs and blood circulation, as well as the therapeutic effect and mechanism analysis of other stem cells on AS.

## Data Availability

Not applicable.
